# A feasibility study of perioperative vitamin D supplementation in patients undergoing colorectal cancer resection

**DOI:** 10.3389/fnut.2023.1106431

**Published:** 2023-03-31

**Authors:** P. G. Vaughan-Shaw, L. F. Buijs, J. P. Blackmur, A. Ewing, H. Becher, E. Theodoratou, L. Y. Ooi, F. V. N. Din, S. M. Farrington, M. G. Dunlop

**Affiliations:** ^1^MRC Human Genetics Unit, Institute of Genetics and Cancer, The University of Edinburgh, Edinburgh, United Kingdom; ^2^Cancer Research UK Edinburgh Centre, Institute of Genetics and Cancer, The University of Edinburgh, Edinburgh, United Kingdom; ^3^Centre for Global Health Research, Usher Institute for Population Health Sciences and Informatics, The University of Edinburgh, Edinburgh, United Kingdom; ^4^Department of Pathology, National University Hospital, Singapore, Singapore

**Keywords:** colorectal < cancer type, vitamin D, nutraceuticals, supplementation, surgery

## Abstract

**Background:**

Vitamin D supplementation improves colorectal cancer (CRC) survival outcomes in randomized trials. The aim of this study was to test the feasibility, safety and efficacy of vitamin D supplementation in the pre- and perioperative period in patients undergoing CRC surgery.

**Methods:**

Patients were given 3200IU oral cholecalciferol (D3) per day perioperatively. Serial serum 25-hydroxyvitamin (25OHD) was measured by liquid chromatography tandem mass spectrometry and compared to untreated CRC controls. 25OHD and C-reactive protein (CRP) levels were compared using adjusted generalized linear mixed-effects models.

**Results:**

A total of 122 patients underwent serial perioperative sampling, including 41 patients given high-dose perioperative supplementation. Supplementation was well-tolerated with no adverse or serious adverse events related to supplementation reported. Pre-operative supplementation increased 25OHD levels on the day of surgery (103.9 vs. 42.5 nmol/l, *P* = 8.2E–12). Supplementation increased 25OHD levels at all post-operative timepoints (*P* < 0.001) and attenuated the post-operative drop in 25OHD (46 vs. 24% drop, *P* = 3.0E–4). Rate of vitamin D peri-operative insufficiency was significantly less in those on supplementation (e.g., day 3–5, 14 vs. 84%, *P* = 1.41E–08), with multivariate modeling across all timepoints indicating a ∼59 nmol/l higher 25OHD compared to control patients (*P* = 3.7E–21). Post-operative CRP was lower in patients taking supplementation (e.g., day 3–5 timepoint; 129 vs. 81 mg/l, *P* = 0.04).

**Conclusion:**

High dose pre-operative vitamin D supplementation is associated with higher perioperative 25OHD levels, lower rates of vitamin D insufficiency and reduced early post-operative CRP. Alongside published evidence for a beneficial effect of vitamin D on CRC survival outcomes, these novel findings provide strong rationale for early initiation of vitamin D supplementation after a diagnosis of CRC.

## Introduction

Despite substantial improvement in mortality from colorectal cancer (CRC) over recent years, ∼16,600 people die from CRC each year in the UK (1). Indeed, the overall global cancer burden is increasing year on year, with ∼10 million deaths per year (1). Whilst surgery remains the mainstay of therapy for the majority of solid cancers, new therapeutic approaches are required. Compelling evidence from observational studies (2) demonstrate a link between low vitamin D and poor outcomes following a diagnosis of colorectal cancer (3–8), while, crucially, a recent meta-analysis of trial data demonstrates a 30% reduction in adverse CRC outcomes with supplementation (9). Plasma 25-hydroxyvitamin D (25OHD) levels in CRC patients are consistently lower than in population controls (10), yet a key unanswered question is whether supplementation could provide benefit from the time of diagnosis. Abdominal surgery is a major physiological insult and 25OHD (the best marker and storage form of vitamin D) falls dramatically following CRC surgery (2). Meanwhile, 25OHD is also known to decrease following orthopedic, cardiac and gynecological surgery (11–16). Suggested explanations include circulatory fluid changes, i.e., hemodilution (14, 17) and/or systemic inflammatory responses to surgery. It follows that because 25OHD depletion is associated with adverse survival outcomes and supplementation can improve survival, studies to define when to initiate supplementation are required. While no study to date has assessed the impact of vitamin D supplements in patients undergoing colorectal cancer resection, cholecalciferol (vitamin D3) supplements have been shown to significantly improve 25OHD levels in patients with a historical diagnosis of CRC (18) and in patients undergoing chemotherapy for CRC (19).

Here, we have investigated the perioperative temporal variation in plasma 25OHD and CRP by serially sampling patients undergoing CRC resectional surgery and aimed to demonstrate that vitamin D supplementation is feasible, safe and effective in the perioperative period. These data will be essential in informing the design of future randomized trials of vitamin D and survival outcomes in patients with colorectal cancer.

## Materials and methods

All participants provided informed written consent, and research was approved by local research ethics committees (SOCCS 11/SS/0109 and 01/0/05; SCOVIDS 13/SS/0248), National Health Service management (SOCCS 2013/0014, 2003/W/GEN/05; SCOVIDS 2014/0058) and registered with www.clinicaltrials.gov (NCT05506696). Clinical and sampling variables were collected from patient case notes and pathology records, entered into a prospective study database and extracted for analysis. We recruited patients undergoing curative colorectal cancer resection to undergo high dose pre- and perioperative oral vitamin D supplementation (3200IU/day cholecalciferol, Fultium-D3, Internis Pharmaceuticals Ltd, Huddersfield, UK), alongside a contemporaneous and historical control group (2). Any patients receiving neo-adjuvant therapy were sampled and provided supplementation after completion of pre-operative oncological therapy. The chosen dose was the maximum available within the hospital formulary and with reference to doses supplied in relevant CRC vitamin D trials (9). All patients over 16 years of age and eligible for supplementation but not already established on vitamin D or multivitamin supplementation were considered for inclusion. Patients were excluded if contra-indicated to high-dose vitamin D supplementation (severe kidney disease, hypercalcemia, hyperparathyroidism, atherosclerosis, sarcoidosis, histoplasmosis, lymphoma, female and child bearing age, or taking thiazide diuretics, digoxin or other cardiac glycosides). Whether the patient was recruited by our research nurse, or surgical research fellows (PVS/LB) determined whether they were allocated to the control or supplementation group, respectively, itself determined by timetabling and clinic availability of respective recruiter. Patients in the supplementation arm took 3200IU cholecalciferol (D3) daily preoperatively, including on the morning of surgery, and in the early post-operative period until discharge except where post-operative ileus occurred. As this was a pragmatic feasibility study we were unable to dictate operative scheduling, and so patients took preoperative supplementation from the pre-operative surgical clinic until their surgery, which in our unit is ∼4 weeks. Supplementation was given in the early post-operative period and discontinued at the point of discharge from hospital. The study protocol allowed a maximum of 12 weeks’ supplementation to be given. Recruitment was paused during the first wave of the COVID-19 pandemic, significantly impacting on total study recruitment, while restrictions on hospital appointments and non-clinical sampling limited the number of post-discharge samples that could be taken. Management and reporting of adverse events and serious adverse events was as per ACCORD (Academic and clinical office for research support, University of Edinburgh) standard operating procedures.

### Plasma vitamin D assay

**FIGURE 1:**
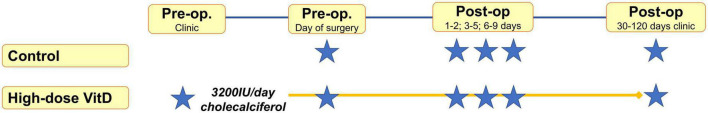
May-adjusted peri-operative 25-hydroxy vitamin D (25OHD) in supplemented and control groups Median 25OHD levels charted in nmol/l. Number of patients in each group and P-values given. False discovery rate (FDR) P-value given adjusted in mixed-effects model for gender, age, body mass index (BMI), cancer site, operative approach, and American Joint Committee on Cancer stage (AJCC).

Samples were taken at preoperative assessment clinic and/or the day of surgery, and post-operatively (on the ward at 1–2, 3–5, 6–9 days) and at the first post-operative clinic appointment (30–120 days, Figure 1). Venepuncture was performed from a peripheral arm vein using a 4 ml S-Monovette^®^ Serum Gel tube (Sarstedt AG & Co., Nümbrecht, Germany), with serum extracted using recommended centrifugation settings of 2,500 × g at 20°C for 10 min. Samples were submitted for 25OHD analysis in batches, with samples from an individual patient analyzed in the same batch to reduce intra-patient variation. Furthermore, to minimize any potential analytical variation in plasma 25OHD measurement, all samples were assayed in a single United Kingdom Accreditation Service (UKAS) accredited laboratory using a method traceable to National Standards of Science and Technology standard reference material (20). Total 25OHD (25OHD2 and 25OHD3) was measured by liquid chromatography tandem mass spectrometry with low coefficient of variation (<10%) in previously published data (20) and analyzed using the Waters^®^ Xevo^®^ TQ-S system (Waters Limited, Wilmslow, UK).

### Plasma CRP assay

To assess for potential relevance of the systemic inflammatory response, samples were assayed for CRP in an NHS Biochemistry Laboratory serving our hospital. CRP was measured using the Abbott Architect C series clinical chemistry analyzer to standard sensitivity protocol, with the range of output values 0.2–480 mg/l. CRP internal quality control was performed daily, with three control samples assayed twice per day. Target mean of the three control samples was 3.2, 8.3, and 27.5 mg/l with actual observed mean over 6 months of 3.2, 8.4, and 27.8 mg/l, respectively, from 5,151 completed assays. Coefficients of variation were 3.42, 2.34, and 2.05%, respectively.

### Sample size considerations

Data from our previously published control group (2) was used to inform a power calculation, with the current study design powered to identify a 50% suppression of the day 1–2 post-operative drop in 25OHD with supplementation [α = 0.05 (one-tailed); β = 0.10, calculated sample size in each *arm* = 54].

### Patient and public involvement

This project is relevant to biomarker prediction and risk stratification and was assigned high priority in the seminal Association of Coloproctology of Great Britain and Ireland (ACPGBI) Delphi process (21). After draft protocol design, we developed our lay summary and PPI questionnaire in collaboration with the University of Edinburgh Patient Advisory Group and Patient Liaison Group of the ACPGBI. Thereafter we surveyed eight patients with colorectal cancer to assess the acceptability of the proposed serial sampling study and help inform our final study design.

### Patient, tumor and treatment-related variables

We considered and adjusted for patient-related factors previously established to influence 25OHD levels [age, sex, body mass index (BMI), neo-adjuvant therapy and adjuvant therapy use, operative approach and cancer site] (22–24). Tumor and treatment-related variables were collected to investigate putative effect on 25OHD, including American Joint Committee on Cancer (AJCC) stage. Information on tumor site, multiplicity and clinico-pathologic staging were obtained from clinical records, along with preoperative imaging. By using collated pathology, imaging, and clinical data; tumor stage was mapped onto the AJCC staging system (AJCC stage I to IV).

### Statistical analysis

All statistical analysis was undertaken in R (version 4.1.0) (25). To account for seasonal differences in vitamin D status, reported 25OHD level was May-standardized by adjusting for sampling month using differences in age- and sex-adjusted monthly averages generated from SOCCS control data. Baseline data in the control and treatment groups were compared using Mann-Whitney, Chi-squared or Fisher’s exact test, with significant difference considered as *P* < 0.05. Baseline 25OHD was compared between groups using a multivariable linear regression model adjusting for gender, AGE, BMI, AJCC, cancer site and operative approach. We also tested for baseline differences between the two control groups in the same way. Next, we used the “*lme4”* package (26) to generate a linear mixed-effects model of the serially sampled data to examine the association between perioperative changes in 25OHD (log-transformed) and treatment group. This multivariable model adjusted for relevant clinico-demographic factors (*gender, age, BMI, AJCC, and operative approach*). Additional multivariable linear mixed-effects analyses assessing the intervention against each control group separately and including neo-adjuvant/adjuvant therapy use were performed and included in Supplementary material. The package “*emmeans”* (27) was used to compute contrasts between estimated marginal means to evaluate adjusted differences in 25OHD between treatment groups at each timepoint. The post-operative drop in 25OHD was calculated as a fold-change between pre- and post-operative values and compared between treatment groups using Mann-Whitney test. Finally, a binomial generalized linear mixed-effects model was applied to test for differences in rate of vitamin D insufficiency between treatment groups at each peri-operative timepoint, adjusting for all relevant clinic-demographic factors. “*emmeans” w*as evaluate adjusted differences in rate of insufficiency between treatment groups at each timepoint.

To investigate effect on CRP level, a linear mixed-effects model was applied to the serially sampled data to examine the association between perioperative changes in CRP (log-transformed) and treatment group adjusting for clinico-demographic factors. As above the package “*emmeans”* (27) was used to evaluate adjusted differences in CRP between treatment groups at each timepoint.

## Results

We recruited a total of 122 patients, including 41 patients who received high-dose perioperative vitamin D supplementation (Table 1). No significant differences in baseline clinicopathological variables between the groups were observed, while no differences between the previously published control group and newly recruited control group were observed, including in baseline 25OHD (46.3 vs. 47.6 nmol/l, *P* = 0.75; Supplementary Table 1).

**TABLE 1:** Clinicopathological demographics of included patients.

	Control	VitD	*P*
*N*	81	41	–
Sampling years	2012–2020	2020	–
Age (years, SD)	66.4 (13.0)	64.7 (14.1)	0.72
Gender (F)	37 (46%)	17 (41%)	0.70
BMI (kg/m^2^, SD)	27.0 (4.4)	28.5 (5.7)	0.11
Baseline 25OHD (median, IQR)	42.5 (31.9)	50.49 (27.79)	0.35
**Cancer site**			0.06
Colon	49 (60%)	17 (41%)	–
Rectum	32 (40%)	24 (59%)	–
**Surgical approach**			–
Open	36 (44%)	13 (32%)	0.18
Minimally invasive^†^	45 (56%)	28 (68%)	–
**Oncological treatment**			–
Neo-adjuvant^‡^	7 (9%)	7 (17%)	0.23
Adjuvant	18 (22%)	16 (39%)	0.05
**Cancer stage**			0.74
AJCC 1	26 (32%)	8* (21%)	–
AJCC 2	27 (33%)	12 (32%)	–
AJCC 3	19 (23%)	17 (45%)	–
AJCC 4	9 (11%)	1 (3%)	−

*Three additional patients who underwent neo-adjuvant therapy classified as ypT0 on histological analysis. ^†^Includes laparoscopic and robotic-assisted surgery. ^‡^Four patients chemo-radiotherapy and three patients short-course radiotherapy in each group.

### Efficacy and safety of perioperative supplementation

**FIGURE 2:**
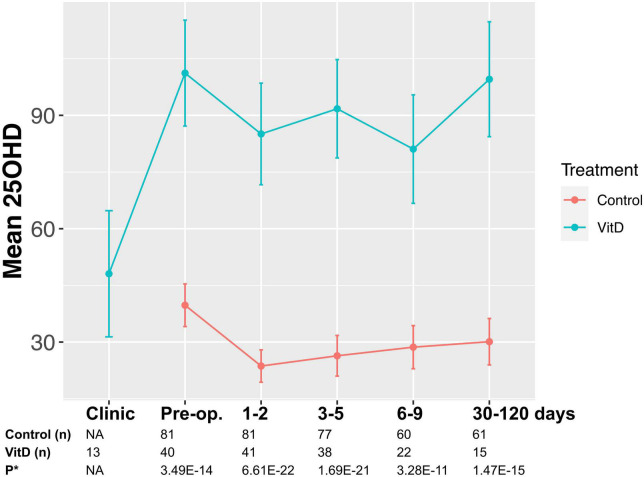
Study flow diagram* indicates sample point. Median sampling days from surgery were comparable between control and supplemented subjects at timepoints 1–2, and 3–5 (*P* > 0.05). However, it was noted that fewer supplemented subjects were sampled at timepoint 6–9 and 30–120 days, with sampling occurring earlier in the supplemented patients (6 vs. 7 days, *P* = 0.04; 39 vs. 67 days, *P* = 0.0001).

No adverse or serious adverse events related to supplementation occurred in those taking perioperative supplementation, which was well-tolerated in study participants. No episodes of hypercalcemia or renal impairment were reported, and no statistically significant changes in calcium or estimated glomerular filtration rate (eGFR) were noted during a previous study of 12 weeks’ supplementation at the same dose (28). Supplementation induced a median 1.9-fold-increase in 25OHD level in the lead up to surgery (48.2, 95% CI 20.7–75.6 nmol/l; *P* = 0.01, Figure 2). Overall, patients took preoperative supplementation for median 25 days (range 9–84). Dose diaries (*N* = 8 subjects), indicated high compliance with daily supplementation (316 out of 319 days perioperative supplementation completed).

Supplemented patients had higher 25OHD levels on the day of surgery (103.9 vs. 42.5 nmol/l, *P* = 8.2E–12, Supplementary Table 2 and Figure 2) and in the early post-operative period (e.g., 1–2 days 80.9 vs. 26.7 nmol/l, *P* = 3.6E–15). Multivariate mixed-effects modeling confirmed a significant and clinically relevant impact of supplementation on perioperative 25OHD levels across all timepoints, after adjusting for age, gender, BMI, cancer site, operative approach and AJCC stage (25OHD estimate 59.4, 95% CI 49.5–70.9 nmol/l, *P* = 3.7E–21, Supplementary Table 3). Supplementation also reduced the prevalence of perioperative vitamin D insufficiency (25OHD<50 nmol/l), with just 14% of supplemented patients having early post-operative insufficiency compared to 84% of control patients (day 3–5 timepoint, *P* = 1.41E–08, Table 2). Finally, although 25OHD levels dropped in all treatment groups following surgery, supplementation attenuated the early post-operative drop (e.g., 46 drop vs. 24% drop at days 1–2, *P* = 3.0E–04) (Figure 2 and Supplementary Table 4).

**TABLE 2:** 25-hydroxyvitamin D (25OHD) insufficiency at each peri-operative timepoint in control and supplemented patients.

Timepoint	Control	High-dose vit D			
	***N* (%)**	***N* (%)**	***P* ^†^**	OR (95% CI)*	*P* *
Pre–op	55 (68%)	3 (8%)	3.06E–09	5.59 (3.40–7.77)	5.37E–07
1–2 days	69 (86%)	11 (28%)	1.37E–10	5.20 (3.23–7.17)	2.18E–07
3–5 days	64 (84%)	5 (14%)	7.45E–14	6.52 (4.27–8.78)	1.41E–08
6–9 days	52 (87%)	4 (20%)	1.04E–07	5.66 (3.3–8.01)	2.52E–06
30–120 days	51 (84%)	1 (7%)	2.29E–09	7.77 (4.36–11.17)	7.92E–06

Vitamin D insufficiency <50 nmol/l. ^†^Univariate *P*-value. *Odds ratio (OR) and false discovery rate (FDR) *P*-value given adjusted in mixed effects model for gender, age, body mass index (BMI), American Joint Committee on Cancer stage (AJCC), cancer site and operative approach.

### Impact of supplementation on post-operative CRP levels

As expected, CRP increased post-operatively in all groups, yet the increase appeared less in those taking high-dose supplementation, with lower early post-operative CRP levels seen in these patients (e.g., 3–5 days 80.5 vs. 129 mg/l, *P* = 0.04, Table 3 and Supplementary Figure 1). On multivariate mixed-effects modeling supplementation had a non-significant impact on peri-operative CRP levels across all timepoints (*P* = 0.07; Supplementary Table 5), with post-operative CRP levels ∼15 mg/l lower in patients on supplementation (15.2, 95% CI 13.8–15.6 mg/l).

**TABLE 3:** Perioperative C-reactive protein (CRP) levels in control and supplemented patients.

	Control		VitD			
**Timepoint**	***N***	**CRP (mg/l, iqr)**	**N**	**CRP (mg/l, iqr)**	***P* ^†^**	*P* *
Pre-op	59	5.0 (11)	38	3.0 (9)	0.16	0.04
1–2 days	58	97.5 (62.25)	39	78.5 (48)	0.049	0.38
3–5 days	57	129.0 (121)	39	80.5 (72.6)	0.017	0.04
6–9 days	46	61.0 (58)	18	54.0 (75)	0.95	0.85

Median C-reactive protein (CRP) levels given in mg/l with inter-quartile range (IQR). ^†^Univariate *P*-value. *False discovery rate (FDR) *P*-value given from multivariable mixed-effects model.

## Discussion

High-dose perioperative vitamin D supplementation is safe and well-tolerated in patients undergoing colorectal cancer surgery. Compared to control patients, supplementation induces a significant increase in perioperative 25OHD levels, a smaller relative drop in 25OHD following surgery and lower rates of post-operative vitamin D insufficiency. Meanwhile, early post-operative CRP levels may be lower in patients on supplementation supporting the role of vitamin D in regulation of inflammatory processes.

This study provides evidence for a beneficial effect of vitamin D supplementation on perioperative vitamin D status while a recent meta-analysis of RCT data reported a significant reduction in CRC mortality with vitamin D supplementation (9, 29). Taken together, these data support early initiation of supplementation at the point of CRC diagnosis. While vitamin D repletion has already been shown to be feasible in patients undergoing chemotherapy (3, 4, 19), no study to date has explored its use in CRC patients in the perioperative period. Vitamin D levels are known to drop following surgery (2), which is confirmed here in all groups. However, we demonstrate that despite the insult of resectional cancer surgery, and inherent changes in gut motility and absorption, perioperative supplementation induces a marked increase in preoperative 25OHD levels and attenuates the drop in 25OHD following surgery. Given the tight physiological autoregulation that exists, it is unlikely that mechanisms that confer the observed beneficial impact of vitamin D on CRC survival occur in a linear manner, but rather that a threshold effect exists. Given that our previous work suggests that a 25OHD threshold of ∼45–50 nmol/l appears to most strongly associate with survival, it is relevant that in the current study supplementation significantly reduced post-operative vitamin D insufficiency (25OHD<50 nmol/l).

To date, no trial has investigated the optimum time to initiate supplementation. Indeed, few of the supplementation trials in CRC patients include patients undergoing curative resection, with one such trial (AMATERASU trial), recruiting patients at the first outpatient visit after surgery (5). It is relevant that in population trials where recruitment and supplementation occur before the diagnosis of incident cases of CRC, a beneficial effect of vitamin D supplementation on survival is still seen. Furthermore, 25OHD levels sampled preoperatively and at the earliest post-operative timepoint (<6 months) are already associated with survival in observational data (2). Therefore, given that a cheap supplement has now been shown to be safe and well-tolerated in the perioperative period, there is compelling rationale to start at the point of diagnosis.

Previous pre-clinical and human intervention studies provide clues to the mechanisms that may underlie the beneficial effect of supplementation on CRC survival and provide no contra-indication to earlier supplementation. 1,25-dihydroxyvitamin D_3_ modulates immune and inflammatory pathway genes in large bowel epithelium (30) and CRC cell lines (31) while oral supplementation induces transcriptomic changes in rectal mucosa that are linked to anti-tumor effects (32). Vitamin D also regulates multiple inflammatory processes both *in vitro* and *in vivo*, including those involved in CRC such as oxidative stress and the cyclooxygenase and NF-kB pathways (33, 34). Effects on inflammatory processes are consistent with the lower post-operative CRP levels in supplemented patients in the current study, supporting the notion that vitamin D might be causally implicated in the SIR response following surgery. This provides further potential mechanism to improved survival outcomes with supplementation given that CRP, an established marker of inflammation, is strongly correlated with CRC survival (35, 36).

The current study has a number of limitations. We acknowledge that supplemented patients recruited in 2020 were compared against a combined control group including patients recruited in 2012. As such, unmeasured historical differences in demographics, lifestyle (e.g., physical activity, dietary vitamin D intake or UV-B exposure), genetic background [e.g., vitamin D receptor or pathway SNPs (37)], exact sampling times, or clinical factors (e.g., neo-adjuvant therapy prior to supplementation and surgery, post-operative recovery, diet and discharge) may confound the observed effect of supplementation. However, we observed no difference in baseline or peri-operative 25OHD between the two control groups. Furthermore, mixed-modeling identified a significant association between supplementation and increased 25OHD level when comparing supplemented patients with the contemporaneous control cohort alone. Despite this, we cannot fully exclude the possibility that differences in perioperative 25OHD levels are due to factors other than supplementation because this was not a randomized study. Next, given the pragmatic methodology, no alteration to the normal patient pathway occurred and there was marked variation in duration of preoperative supplementation between patients, in part due to delays and disruption from the COVID-19 pandemic which also impacted total study recruitment and sampling. Heterogeneity in exact sampling day within the respective timepoints between the treated and control subjects, driven by differences between the contemporary and historical cohorts is also acknowledged, yet we do not believe that these factors would impact our conclusions given the magnitude and significance of differences in 25OHD levels between supplemented and control patients. Furthermore, given the immediate post-operative drop in 25OHD that is observed across all patients, early sampling in the supplemented patients would be expected to deflate, rather than inflate observed differences with the control cohort. Next, we did not record compliance with post-operative supplementation in regards to return to oral intake or complications precluding oral supplementation. We did not collect albumin or vitamin D binding protein (DBP) levels in this study. We acknowledge such levels may impact levels of available 1,25-dihydroxyvitamin D_3_ (38), the active form of 25OHD. Meanwhile, there is debate surrounding which assay provides the best marker of vitamin D status (39, 40). Given the marked changes in circulating 25OHD observed with supplementation, we would not expect any material changes in the observed differences in our results or conclusions after adjustment for albumin or DBP level. We acknowledge that efforts to assay 1,25OHD in target tissue (i.e., colon or rectum) would be of value in future mechanistic studies. Also, we have reported CRP as an easily assayed and recognized marker of the systemic inflammatory to surgery but acknowledge that this is a non-specific marker. Other markers of inflammation, including pro-calcitonin and interleukins, may provide fuller understanding of specific inflammatory responses to supplementation, surgery or CRC itself. Finally, while our data supports the early initiation of supplementation in patients undergoing CRC surgery given its effects on 25OHD level and extrapolating from previous trial evidence of survival benefit, we do not provide direct evidence of survival benefit from early supplementation, nor do we consider other clinical outcomes (e.g., post-operative morbidity), or patient reported outcomes (e.g., quality of life). Future trials must consider such outcomes in the context of clinically relevant patient benefit.

In conclusion, we report for the first time on the feasibility and safety of perioperative vitamin D supplementation in patients undergoing colorectal cancer surgery. We identified a positive effect of supplementation on perioperative 25OHD levels with lower rates of post-operative vitamin D insufficiency and reduced early post-operative CRP. Our findings provide compelling rationale for early initiation of vitamin D supplementation after a diagnosis of CRC. Future randomized trials of supplementation with a defined endpoint of a beneficial effect on survival outcomes should consider supplementation from the point of diagnosis.

## Data availability statement

The raw data supporting the conclusions of this article will be made available by the authors, without undue reservation.

## Ethics statement

The studies involving human participants were reviewed and approved by South East Scotland Regional Ethics Committee 01, (SOCCS 11/SS/0109 and 01/0/05; SCOVIDS 13/SS/0248). The patients/participants provided their written informed consent to participate in this study.

## Author contributions

PV-S, LB, JB, LO, FD, SF, and MD: study concepts. PV-S, LB, LO, JB, ET, SF, and MD: study design. PV-S, LO, LB, and JB: data acquisition. PV-S and AE: quality control of data and algorithms. PV-S, AE, and HB: data analysis and interpretation and statistical analysis. PV-S, LB, JB, AE, HB, SF, and MD: manuscript preparation and editing. PV-S, ET, AE, FD, SF, and MD: manuscript review. PV-S, SF, and MD: funding acquisition. MD: project administration. MD and SF: supervision. All authors contributed to the article and approved the submitted version.
