# Supplementary material for: A feasibility study of perioperative vitamin D supplementation in patients undergoing colorectal cancer resection
Source: Front Nutr. 2023 Mar 31;10:1106431. doi: 10.3389/fnut.2023.1106431 (PMC10103841; doi:10.3389/fnut.2023.1106431)
Supplement: Supplementary file 1 [file Data_Sheet_1.docx]

**Supplementary Tables and Figures**

**SUPPLEMENTARY TABLE 1 Clinicopathological demographics of included patients stratified by control group sampling years**

|  | **Control A** | **Control B** | **P*** |
| --- | --- | --- | --- |
| N | 62 | 19 | - |
| Sampling Years | 2012-2013 | 2020 | - |
| Age (mean, SD) | 67.5 (12.7) | 60.0 (13.8) | 0.14 |
| Gender (F) | 29 (47%) | 8 (42%) | 0.80 |
| BMI (kg/m^2^, SD) | 27.24 (4.6) | 27.20 (3.9) | 0.59 |
| Baseline 25OHD  (mean, 95%CI)) | 46.3  (39.8-52.9) | 47.6  (34.9-60.3) | 0.75† |
| Cancer site |  |  | 0.43 |
| Colon | 39 (63%) | 10 (53%) | - |
| Rectum | 23 (37%) | 9 (47%) | - |
| Operative approach |  |  | 0.06 |
| Open | 28 | 8 | - |
| Minimally-invasive | 34 | 11 | - |
| Cancer stage |  |  | 0.09 |
| AJCC 1 | 19 (31%) | 7 (37%) | - |
| AJCC 2 | 25 (40%) | 2 (11%) | - |
| AJCC 3 | 12 (19%) | 7 (37%) | - |
| AJCC 4 | 6 (10%) | 3 (16%) | - |

**P value given for univariate tests using Mann-Whitney test for age, BMI and Fisher’s exact or Chi-squared test for categorical variables.* † *for 25OHD comparison between control groups using multivariable linear regression model adjusting for gender, AGE, BMI, AJCC, cancer site and operative approach to confirm baseline 25OHD was the same between the two groups.*

**SUPPLEMENTARY TABLE 2 May-adjusted perioperative 25OHD level in control and supplemented patients**

|  | Control | | High-dose VitD | |  |  |  |
| --- | --- | --- | --- | --- | --- | --- | --- |
|  | **N** | **25OHD** | **N** | **25OHD** | **P†** | **ratio* (95%CI)** | **P*** |
| Clinic | - | - | 13 | 50.5 (27.8) | NA | NA | NA |
| Pre-op | 81 | 42.5 (31.9) | 40 | 103.9 (49.5) | 8.2E-12 | 2.41 (1.94-3.00) | 3.49E-14 |
| 1-2 days | 81 | 26.7 (20.7) | 41 | 80.9 (39.6) | 3.6E-15 | 3.18 (2.56-3.94) | 6.61E-22 |
| 3-5 days | 77 | 28.5 (22.3) | 38 | 92.9 (33.9) | 2.9E-13 | 3.18 (2.55-3.96) | 1.69E-21 |
| 6-9 days | 60 | 28 (26.6) | 22 | 82.5 (36.2) | 1.1E-06 | 2.40 (1.87-3.09) | 3.28E-11 |
| 30-120 days | 61 | 34.2 (21.5) | 15 | 112 (31.2) | 9.4E-08 | 3.15 (2.40-4.14) | 1.47E-15 |

*Median 25OHD levels given in nmol/l with IQR. Pre-op timepoint taken on day of surgery. † univariate P value using Mann-Whitney test. *ratio between control groups and FDR P value taken from multivariable mixed-effects model for gender, AGE, BMI, cancer site, operative approach, and AJCC. To obtain this P value, the package ‘emmeans’ was used to compute contrasts between estimated marginal means to evaluate potential differences in 25OHD between treatment groups at each timepoint.*

**SUPPLEMENTARY TABLE 3 Multivariate mixed-effects model for factors associated with peri-operative 25OHD level.**

| Factor | Estimate | P |
| --- | --- | --- |
| Treatment group |  |  |
| High-dose VitD | 1.04 | 3.70E-21 |
| Clinicodemographic factors |  |  |
| Gender (M) | 0.07 | 0.40 |
| Age | -0.002 | 0.53 |
| BMI | -0.02 | 0.02 |
| AJCC | -0.03 | 0.43 |
| Operative approach (open) | -0.037 | 0.67 |
| Cancer site (rectum) | 0.08 | 0.37 |

*Estimate for impact of supplementation equates to 59.4nmol/l, 95%CI 49.5-70.9nmol/l.If control groups were entered individually the significant impact of supplementation remained (versus 2016 control group P=1.2E-14; versus 2020 control group P=4.46E-08). When use of neo-adjuvant or adjuvant therapy use included in model, significant impact of supplementation remained (P=6.9E-20).*

**SUPPLEMENTARY TABLE 4 Post-operative 25OHD fold-change compared to day of surgery pre-operative level**

|  | Control | High-dose VitD | |
| --- | --- | --- | --- |
| Timepoint | **FC (iqr)** | **FC (iqr)** | **P** |
| Day 1-2 | 0.54 (0.38) | 0.76 (0.27) | 0.0003 |
| Day 3-5 | 0.56 (0.44) | 0.9 (0.29) | 0.0001 |
| Day 6-9 | 0.63 (0.42) | 0.78 (0.59) | 0.17 |

*Median fold-change in unadjusted 25OHD given (iqr). *P value given for univariate Mann-Whitney test*

**SUPPLEMENTARY FIGURE 1 Peri-operative CRP levels**

**
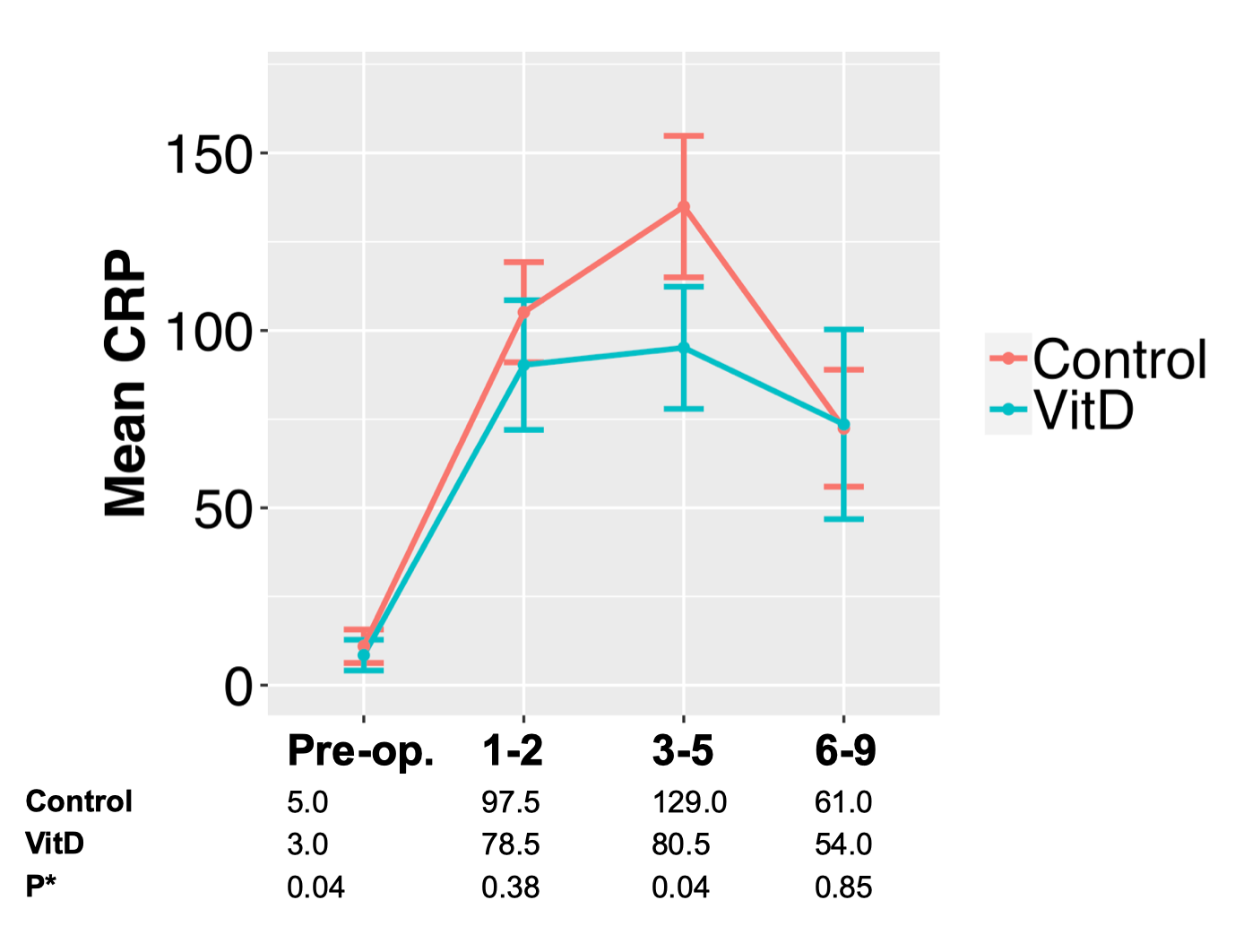
**

**FDR P value given adjusted in mixed effects model for gender, AGE, BMI, AJCC and operative approach*.

**SUPPLEMENTARY TABLE 5 Multivariate mixed-effects model for factors associated with peri-operative CRP level**

| Factor | Estimate | P |
| --- | --- | --- |
| Treatment group |  |  |
| High-dose VitD | -0.22 | 0.068 |
| Clinicodemographic factors |  |  |
| Age | 0.01 | 0.048 |
| Gender (M) | 0.29 | 0.01 |
| BMI | 0.02 | 0.03 |
| AJCC | 0.08 | 0.15 |
| Operative approach (open) | 0.17 | 0.14 |
